# Interfaces Based on Laser-Structured Arrays of Carbon Nanotubes with Albumin for Electrical Stimulation of Heart Cell Growth

**DOI:** 10.3390/polym14091866

**Published:** 2022-05-02

**Authors:** Alexander Yu. Gerasimenko, Evgeny Kitsyuk, Uliana E. Kurilova, Irina A. Suetina, Leonid Russu, Marina V. Mezentseva, Aleksandr Markov, Alexander N. Narovlyansky, Sergei Kravchenko, Sergey V. Selishchev, Olga E. Glukhova

**Affiliations:** 1 Institute of Biomedical Systems, National Research University of Electronic Technology MIET, Shokin Square 1, Zelenograd, 124498 Moscow, Russia; kurilova_10@mail.ru (U.E.K.); selishchev@bms.zone (S.V.S.); 2 Institute for Bionic Technologies and Engineering, I.M. Sechenov First Moscow State Medical University, Bolshaya Pirogovskaya Street 2-4, 119991 Moscow, Russia; markov@bms.zone (A.M.); glukhovaoe@info.sgu.ru (O.E.G.); 3 Scientific-Manufacturing Complex “Technological Centre”, Shokin Square 1, Zelenograd, 124498 Moscow, Russia; kitsyuk.e@gmail.com; 4 World-Class Research Center “Digital Biodesign and Personalized Healthcare”, Sechenov First Moscow State Medical University, 119991 Moscow, Russia; 5 National Research Center for Epidemiology and Microbiology Named after the Honorary Academician N.F. Gamaleya, Gamaleya Street 18, 123098 Moscow, Russia; ikas@inbox.ru (I.A.S.); plano77@bk.ru (L.R.); marmez@mail.ru (M.V.M.); narovl@yandex.ru (A.N.N.); 6 National Medical Research Center for Hematology, Novy Zykovsky Proezd 4, 125167 Moscow, Russia; skkrav@mail.ru; 7 Department of Physics, Saratov State University, Astrakhanskaya Street 83, 410012 Saratov, Russia

**Keywords:** arrays of carbon nanotubes, albumin, laser structuring, electrical stimulation, scanning electron microscopy, Raman spectroscopy, growth of cells, heart cells, bioelectronics

## Abstract

Successful formation of electronic interfaces between living cells and electronic components requires both good cell viability and performance level. This paper presents a technology for the formation of nanostructured arrays of multi-walled carbon nanotubes (MWCNT) in biopolymer (albumin) layer for higher biocompatibility. The layer of liquid albumin dispersion was sprayed on synthesized MWCNT arrays by deposition system. These nanostructures were engineered using the nanosecond pulsed laser radiation mapping in the near-IR spectral range (λ = 1064 nm). It was determined that the energy density of 0.015 J/cm^2^ provided a sufficient structuring of MWCNT. The structuring effect occurred during the formation of C–C bonds simultaneously with the formation of a cellular structure of nanotubes in the albumin matrix. It led to a decrease in the nanotube defectiveness, which was observed during the Raman spectroscopy. In addition, laser structuring led to a more than twofold increase in the electrical conductivity of MWCNT arrays with albumin (215.8 ± 10 S/m). Successful electric stimulation of cells on the interfaces with the system based on a culture plate was performed, resulting in the enhanced cell proliferation. Overall, the MWCNT laser-structured arrays with biopolymers might be a promising material for extended biomedical applications.

## 1. Introduction

The development of biocompatible materials with specific properties represents an important issue in biomedical research [1,2]. In this context, biocompatible conducting materials are of special interest, e.g., for biomedical applications ranging from tissue engineering, drug delivery, and bioimaging, to biosensing [3,4,5,6,7,8,9,10,11,12]. Particularly, tissue engineering with electrical stimulation allows to regulate various cellular behaviors such as cellular adhesion, alignment, proliferation, or differentiation, and thus facilitate the regeneration of damaged tissues, such as skin, bone, nerve, or myocardium tissues [13,14,15,16,17,18,19].

Carbon nanotube (CNT) is a unique material that provides an opportunity to significantly enhance conductivity of the material while remaining its biocompatible properties [20,21,22]. CNTs have a number of useful properties, such as high tensile strength, elasticity, thermal conductivity [23], electrical conductivity [24], and biocompatibility [25]. These properties indicate that the use of CNTs as a part of nanointerfaces for biomedical devices can be promising. These interfaces may be used both for solving diagnostic problems, e.g., reading signals from tissues with high resolution, and for transmitting electrical impulses to cells [26]. It is known that electrical stimulation improves cell differentiation and proliferation [27]. Ionic currents and electrical potentials inside cells affect the function and development of cells and tissues [28]. Disruption or alteration of ionic gradients or surface charges of cells via an applied electric field can lead to changes in cell signaling pathways and gene expression, which lead to positive changes in differentiation, proliferation, and cell motility [29]. Electrical stimulation of cells results in accelerated cell growth, improved cell adhesion, enhanced interaction between cells, and increased cell viability [30,31,32].

One of the important applications of electrically conductive nanointerfaces in biomedicine is the treatment of cardiovascular diseases (CVD), which are currently the leading cause of death in the world [26,33]. An acute shortage of donor organs, along with an increasing demand for them, prompts researchers to look for alternative ways of treating CVD [34]. Currently, there are two main approaches: injection of hydrogels with cells, and implantation of patches with cells and active substances [35]. The disadvantage of both approaches is the low survival rate of the implanted cells, while the additional electrical stimulation can increase their positive outcome rate. When developing an electrically conductive nanointerface, it is necessary to ensure high reproducibility of characteristics and, using a series of preliminary experiments, to select the optimal mode of cell stimulation that improves their survival and does not lead to death [36]. The choice of materials that make up the nanointerface is also important; they must have high biocompatibility and suitable mechanical characteristics [37]. In order to enhance an effect from the electrical stimulation, the alignment of CNT arrays can be performed [38]. In addition, aligned arrays of CNTs promote orientation of cells in one direction, which has a positive effect on the transmission of signals between them, which is especially important for ensuring contractions of the heart tissue [39]. There are a large number of methods for orientation of CNT arrays [40,41,42]. However, preference is given to optical methods, which make it possible to change the structure and shape of CNTs upon contactless exposure to an electromagnetic field [43,44,45,46]. When CNT arrays are treated with laser radiation, bonds are created between the walls and ends of CNTs, due to which it is possible to achieve a decrease in contact resistance and, as a result, higher electrical conductivity of structures and efficient transmission of electrical impulses to cells [47]. In this case, the surface morphology, structure, and CNT binding quality can vary with changes in the amount of energy reported due to changes in the wavelength, power, intensity, and time of irradiation [48].

At the same time, covering the multi-walled carbon nanotube (MWCNT) arrays with biocompatible polymers can improve cell survival in the area of contact with MWCNTs and contribute to a more physiological transition from the hard surface of MWCNTs to the soft surface of human tissues [49,50]. One suitable polymer for this purpose is albumin, an important transport protein in the blood that provides many nutrients for cells [51]. Albumin coatings are often used in cardiovascular engineering to reduce the risk of thrombosis [52]. During laser treatment, albumin amino acids adhere firmly to nanotubes, which increases the stability of the material [53].

It is known that it is possible to create nanocomposites from disordered systems of carbon nanotubes in the albumin matrix, which are formed by evaporation of water–albumin dispersion with nanotubes by laser radiation [53].

It has been established that nanotubes are functionalized by oxygen atoms of negative residues of aspartic and glutamic amino acids, which are located on the outer surface of the albumin molecule [54]. There are two possible types of interactions between nanotubes and biopolymer in a nanocomposite: (1) hydrophobic interaction and (2) formation of covalent bonds. In this regard, there is a significant number of defects in nanotubes caused by covalent attachment of oxygen to the graphene surface. Under the laser radiation, nanotubes form contacts with each other, providing an electrically conductive network [55]. However, creating a homogeneous liquid dispersion with uniformly distributed carbon nanotubes is an extremely difficult task [56,57]. Therefore, such a disadvantage relates to the distribution of nanotubes inside a solid nanocomposite.

In this regard, attractive methods for obtaining planar nanocomposites with a uniformly distributed system of nanotubes will be the methods related to the use of initial nanotubes that are firmly attached to the substrate and ordered.

Therefore, in this article we have proposed a technology for the formation of electrically conductive materials based on vertical MWCNT arrays grown at a silicon substrate, which were ordered and densely distributed over the substrate area.

To obtain biopolymer materials with contacts between nanotubes, vertical arrays, as in [55], were coated with albumin and subjected to laser exposure. The morphology of the obtained materials was characterized using electron microscopy, energy-dispersive spectroscopy, and Raman spectroscopy. The effect of laser radiation energy density on the morphology and electrical conductivity of nanomaterials was also described. With the help of the developed installation, the applicability of the formed nanomaterials as interfaces for electrical stimulation of the growth of connective and cardiac tissue cells has been proved.

## 2. Materials and Methods

### 2.1. Formation of Samples from MWCNT Arrays with Albumin

Arrays of multi-walled carbon nanotubes were first synthesized to form biocompatible interfaces. MWCNT synthesis was carried out according to the following procedure. N-doped 4,5 ohm-cm, (100) oriented silicon 4-inch wafer (Si-Mat, Landsberg am Lech, Germany) was cut to samples with a size of 10 mm × 10 mm. A catalytic pair of Ti (10 nm) and Ni (2 nm) was deposited on a substrate treated in a Piranha solution by the electron beam evaporation method. MWCNT arrays were synthesized by catalytic plasma-enhanced chemical vapor deposition using Nanofab 800 Agile setup based on the platform PlasmaLab System 100 (Oxford instruments, Abingdon, UK). This process was followed by the redox annealing in order to form catalyst nanoparticles on a substrate. The annealing parameters were identical: oxidation at 280 °C for 5 min in O_2_ and Ar with RF plasma of 100 W; reduction at 700 °C for 5 min in NH_3_ and Ar with RF plasma of 100 W. The temperature rise time was 15 min. All stages were carried out in a single cycle without breaking the vacuum. After the synthesis, the samples were cooled down to 280 °C within 30 min without the samples extraction, and then a hydrophilic treatment was performed. The synthesis parameters are presented in Table 1.

To create a biocompatible interface, the MWCNT array was placed in a matrix of bovine serum albumin (BSA). For this purpose, a suspension of distilled water and BSA (99% purity, BioClot, Aidenbach, Germany) with a concentration of 5 wt.% was prepared. The BSA suspension was layered onto the MWCNT arrays using the spray deposition method. To perform this, a modified E2V dosing system (Nordson EFD, Westlake, OH, USA) with an outlet nozzle diameter of 0.5 mm was used. The distance between the sprayer and the substrate was 10 cm. The suspension supply pressure was 2 bar. The samples were placed on a heating table to evaporate water at temperatures up to 60 °C. Up to 50 layers were applied to fully cover the MWCNT arrays.

### 2.2. Laser Structuring

**Figure 1:**
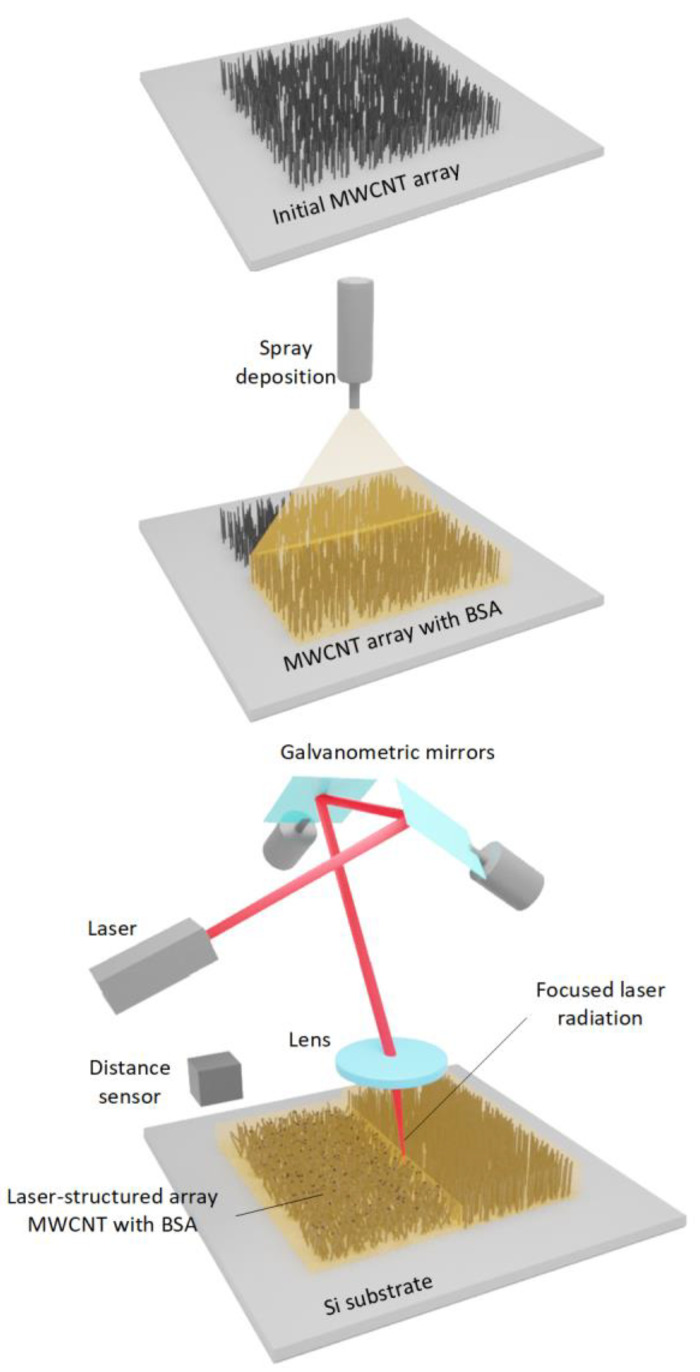
Manufacturing process of electrically conductive interfaces.

To increase the electrical conductivity, MWCNT arrays were structured by exposure to laser radiation. For this, a pulsed ytterbium fiber laser with a wavelength of 1064 nm (pulse duration of 100 ns and pulse repetition rate of 100 kHz, power of up to 0.13 W) was used. A galvanometric scanner with two mirrors (Figure 1) was used to position the laser beam over the sample area.

Then, the beam reached the objective, which focused the irradiation on the sample surface into a 35 µm spot. The laser mapping step was about 100 μm. The energy density ranged from 0.001 to 0.017 J/cm^2^. The optical setup was equipped with a distance sensor for uniform irradiation of the sample area.

For the laser processing of samples, the scanning parameters were set via a specialized software. The beam moved along a trajectory describing a square, which was filled with lines with a slight overlap of laser spots on each other. This was performed in order to compensate the intensity of the laser’s Gaussian beam profile. The beam movement speed along the trajectory was 500 mm/s. The laser pulse lines were 5 mm long, parallel to each other at a distance of 17 μm (Figure 1).

Thus, research included the following groups: 1. Si substrates with a synthesized MWCNT array; 2. Si substrates with MWCNT array with BSA coating; 3. Si substrates with a laser-structured MWCNT array with BSA coating at different radiation energies. Each group contained five samples to obtain statistical results when reexamined. A substrate of pure oxidized silicon was used for control.

### 2.3. Electron Microscopy

The study of samples’ topological features on Si substrates was carried out using scanning electron microscopy (SEM) with FEI Helios NanoLab 650 (FEI Ltd., Hillsboro, OR, USA). Accelerating voltage of electron column was 5 kV, and electron probe current was 50 pA for MWCNT arrays. For samples coated with BSA, accelerating voltage and electron probe current was 1 kV and 25 pA, correspondingly. Pressure in the vacuum chamber was 3.9 × 10^−4^ Pa. The samples were attached to a conductive substrate using a carbon tape. To study the morphological features of MWCNTs, transmission electron microscopy (TEM) was used with a JEOL JEM-2100Plus complex at an accelerating voltage of an electron column of 200 kV.

For this purpose, MWCNTs were mechanically transferred to a copper meshes. To determine the size of the MWCNTs, distance measurements were carried out using the microscope Control software. A total of 15 repetitions were performed for each measurement.

### 2.4. Raman Spectroscopy

Raman spectroscopy is an effective tool for monitoring the structure of carbon nanotubes [58]. Since there is a high probability of lattice defects (vacancies), bends, and curvatures within the synthesis of vertical MWCNT arrays, Raman spectroscopy is an effective method for detecting such defects. Raman spectra of nanotube arrays contain scattering bands G and D. With an increase in the nanotube defectiveness, the intensity of the D-band increases compared to the G-band, since when bonds are broken in the lattice, atoms with sp^3^-hybridized electrons appear outside the plane of the nanotube layers [59]. Thus, the ratio of the intensities of the D and G bands is useful as a criterion for the defectiveness of the structure based on MWCNT arrays. Sample spectra were obtained in backscattering geometry on a LabRAM HR Evolution (Horiba Ltd., Villeneuve-d’Ascq, France). The spectra were excited by an Ar laser (wavelength 514 nm, power 0.125 mW). The 1800 gr/mm diffraction grating provided a spectral resolution of 0.5 cm^−1^. A BX41 microscope (Olympus Corp., Tokyo, Japan) and a precision motorized table were used to focus the laser beam on the study area. The signal accumulation time was 15 s with averaging over 3 spectra to improve the signal-to-noise ratio.

### 2.5. Electrical Measurements

We analyzed the samples’ resistance via four-probe measurement technique using a multimeter (34401A, Keysight Technologies Inc., Santa Rosa, CA, USA). The resulting resistance value averaged over several experiments was further evaluated into specific conductivity, considering the geometric dimensions of the sample, using the following equation:

σ = 1/r,(1)

where σ and r are the conductivity and the resistivity of the samples, respectively.

### 2.6. Fibroblast Cell Culture on the Samples

Fibroblast cell line (FH-T), which was acquired at the cell culture collection of the National Research Center for Epidemiology and Microbiology of the Ministry of Health of the Russian Federation, was used. Cells were cultured in Dulbecco’s Modified Eagle Medium (DMEM), 90% culture medium supplemented with 10% calf fetal serum in a 6-well plate. The samples for the cell line were prepared under sterile conditions. Directly after preparation, they were irradiated with ultraviolet light for 20 min. Immediately prior to the incubation, samples were rinsed in DMEM for 10 min to eliminate contaminants. Then, samples were placed on the bottom of the wells of a 6-well culture plate, and a few milliliters of the medium were poured into each well with the sample. After being kept for a few seconds, the medium was removed. Finally, the medium with cells was filled in. Determination of the exact number of cells taken in the experiment was carried out immediately before seeding the freshly prepared culture mixture in the wells with the sample, using an automatic cell counter (Scepter Millipore, Merck KGaA, Darmstadt, Germany). The FH-T cell concentration was approximately ≈ 4 × 10^4^ cells/mL. Cells were incubated for 24 h in a thermostat with 5% CO_2_ at 37 °C. Further, under similar conditions of the thermostat, electrical stimulation of cells was carried out for 48 h.

### 2.7. Cardiomyocytes Cell Culture on the Samples

Cardiomyocytes were obtained from the heart of a Wistar rat with the permission of the local ethics committee of the National Research Center for Epidemiology and Microbiology of the Russian Federation Ministry of Health. The rats were up to 3 months old. The rats were placed in a glass container with gauze soaked in chloroform and the monitoring of their condition was performed. After 40 min, the rats were placed into a state of anesthesia.

The selected material was transferred into a sterile Petri dish. Using scissors and tweezers, they were dissected to pieces of 4–7 mm in size and washed twice with phosphate-buffered saline to remove mucus and blood elements. Then, the fragments of the tissue were poured with 0.25% trypsin solution at a temperature of 37 °C, and a sterile magnetic anchor was inserted into the flask. Then, the detached cells, together with trypsin, were poured into centrifuge tubes and placed in an ice cuvette to limit further action of trypsin on the cells. A second portion of trypsin was added to the remaining tissue, and the flask was placed on a magnetic stirrer. The rotation speed was adjusted in such a way to avoid foaming of the contents of the flask. Trypsinization was performed 4 times, but not until the tissue was completely depleted.

After the end of trypsinization, the resulting cell suspension was centrifuged at 1000 rpm for 20 min. The supernatant was decanted, and the cell pellet was resuspended in DMEM medium with 10% fetal bovine serum at 37 °C and poured into a flask through a filter. After stirring the cell suspension, two samples of 0.5 mL were taken. To each sample, 0.5 mL of 0.1% crystal violet in citric acid solution was added and then stirred. Then, a drop of the suspension was placed in the Goryaev counting chamber and the number of cells with a distinguishable nucleus and intact cytoplasm was counted. Fetal bovine serum and antibiotic were added to the nutrient medium. Removal of culture during subculture was performed on the 5th day. Culture flasks (mattresses) with cells were examined under a microscope at 70× magnification and selected with a full monolayer and a transparent medium. Viable cells had clear boundaries and typical morphology without pronounced granularity and vacuolization.

The procedure for planting cardiomyocytes on a sample and culturing them was similar to the above-mentioned procedure for fibroblasts.

### 2.8. Stimulation with Electric Field

**Figure 2:**
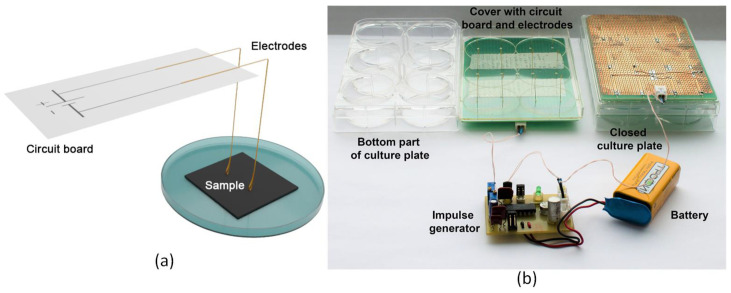
Schematic view (**a**) and photograph (**b**) of experimental setup for electrical stimulation of cell growth.

Figure 2 shows a developed experimental setup for electrical stimulation of cell growth. Acupuncture sterile needles made of gold-plated surgical steel with a diameter of 0.3 mm were used as electrodes in the electrostimulation device. Acupuncture needles are widely used in a medical practice. The material they are made of has a high degree of biocompatibility with the human body, as well as the electrical conductivity that is necessary for high-enough conducting electrical impulses. The needles were bent at a 90° angle. Figure 2a schematically represents the position of the electrodes soldered to the breadboard relative to the samples. The tip of the needle adjacent to the sample was also bent, forming a loop-like shape. This was necessary to smooth the contact between the needle and the sample. The other end was soldered to the breadboard, allowing the electrode to spring into contact with the substrate’s surface. Each well of culture plate contained two electrodes (positive and negative); the distance between them was 24 mm. In the lid of the culture plate, two holes with a diameter of 0.4 mm were formed. Their lo-cation corresponded to the position of the electrodes passing through the holes in the plate.

The electrical stimulation setup consisted of a culture 6-well plate, electrodes, an electric pulse generator, a power supply, and connection elements (i.e., electrical wires and clamps) (Figure 2b).

As a power supply, a “Krona (6F22)” type rechargeable manganese–zinc salt battery with a nominal voltage of 9 V was used. During the electrostimulation, all of the setup elements were placed in the CO_2_ incubator. The incubator provided the necessary environmental conditions (i.e., the temperature of 37 °C and the carbon dioxide content of 5%) for the vital cells activity.

Based on MTT assay, it was found that optimal electrical stimulation of cell growth was provided by an electrical pulse generator, producing a continuous sequence of electrical pulses of 80 µV amplitude. The pulse duration was 2.5 ms, and pause duration was 1.2 s.

### 2.9. Cell Viability Study

Live cell staining was performed using 10 mg/mL fluorescein diacetate and 5 µg/mL ethidium bromide (both Sigma-Aldrich, Darmstadt, Germany) in a supplemented cell growth medium to stain mainly cells nuclei. Cells with fluorescein diacetate were incubated for 15 min at 37 °C, and then with ethidium bromide for 5 min at 37 °C. The live imaging was conducted at day in vitro (DIV) 1. The imaging was performed via a fluorescence microscope (Olympus BX43, Olympus Corporation, Tokyo, Japan) and via a laser scanning microscope (LSM880, Carl Zeiss, Berlin, Germany) using the Zen software (Zen 2.3 SP 1 FP3 Black, Carl Zeiss Microscopy GmbH, Jena, Germany). In each case, three images were acquired. Pure silicon substrates without MWCNTs and cover glasses were used for control samples. In addition, the MTT assay was used to assess cell viability [60]. MTT analysis was performed by determining the optical density on a microplate photometer (Immunochem-2100, High Technology Inc., North Attleboro, MA, USA). The optical density was proportional to the number of living cells. The culture medium was removed from the wells of the tablet after the study. A total of 100 mL of pure nutrient medium and 20 mL of MTT at a concentration of 5 mg/mL were added to each well. Then, the cells with MTT were kept for 2 h in a CO_2_ thermostat. Next, the culture medium and MTT were removed, and 100 mL of dimethyl sulfoxide was added. This was necessary for the dissolution of formazane, which was restored by cells. Then, the cellular sediment was resuspended for 10 min and the optical density was measured at a wavelength of 492 nm. Cell viability was calculated as the percentage of cells grown on nanocomposites in relation to the number of cells grown in the control.

## 3. Results and Discussion

For the analysis of the MWCNT interface quality change after the laser structuring and its biocompatibility (i.e., enhanced cell metabolism and cell functionality) with and without electrical stimulation of cells, an in-depth investigation of the synthesized arrays was performed. The morphology, which correlates to the surface quality of the resulting arrays, was tested via SEM, TEM, and Raman spectroscopy. Electron microscopy is rather illustrative since it precisely provides a visualized proof of the qualitative surface enhancement of MWCNT arrays. Moreover, these experiments (SEM, TEM, and Raman spectroscopy) are well established for the characterization of the CNTs in general. Furthermore, the influence of laser structuring was analyzed together with electrical stimulation to investigate the resulting cell proliferation. Herein, fluorescence microscopy measurements provide qualitative and quantitative analysis.

### 3.1. Morphology of Interfaces Based on MWCNT Arrays with BSA

**Figure 3:**
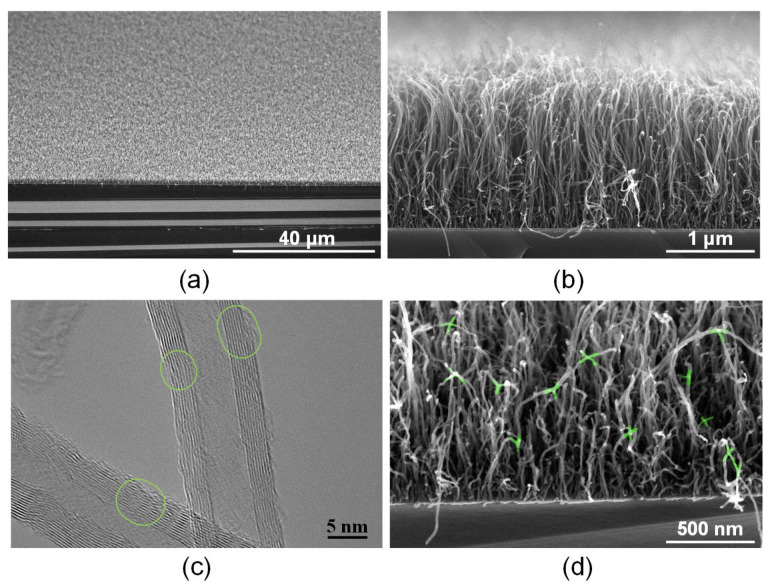
SEM (**a**,**b**) and TEM (**c**) images of MWCNT arrays on Si substrate before and after laser exposure with an energy density of 0.013 J/cm^2^ (**d**). Green ovals show transition regions in which there is a higher number of defects in the area compared to that in the defect-free part of the nanotube framework. Nanotube binding regions are highlighted in green.

The synthesized MWCNT arrays were an ordered system of vertically aligned carbon nanotubes. The average diameter was determined mainly by the size of the catalyst nanoparticles formed on the substrate. The height of the array was ~2 microns (Figure 3a,b). TEM images of nanotubes show the structure of individual multilayer nanotubes (Figure 3c). The nanotubes had an outer diameter of 12–15 nm, the number of walls was 8–15, the wall thickness was 3–5 nm, and the diameter of the inner channel was 4–7 nm. Defects were present on the walls of the nanotubes, including outer walls [61,62]. Figure 3 marks, with green ovals, transition regions in which there is a lower number of defects in the area compared to that in the defect-free part of the nanotube framework.

**Figure 4:**
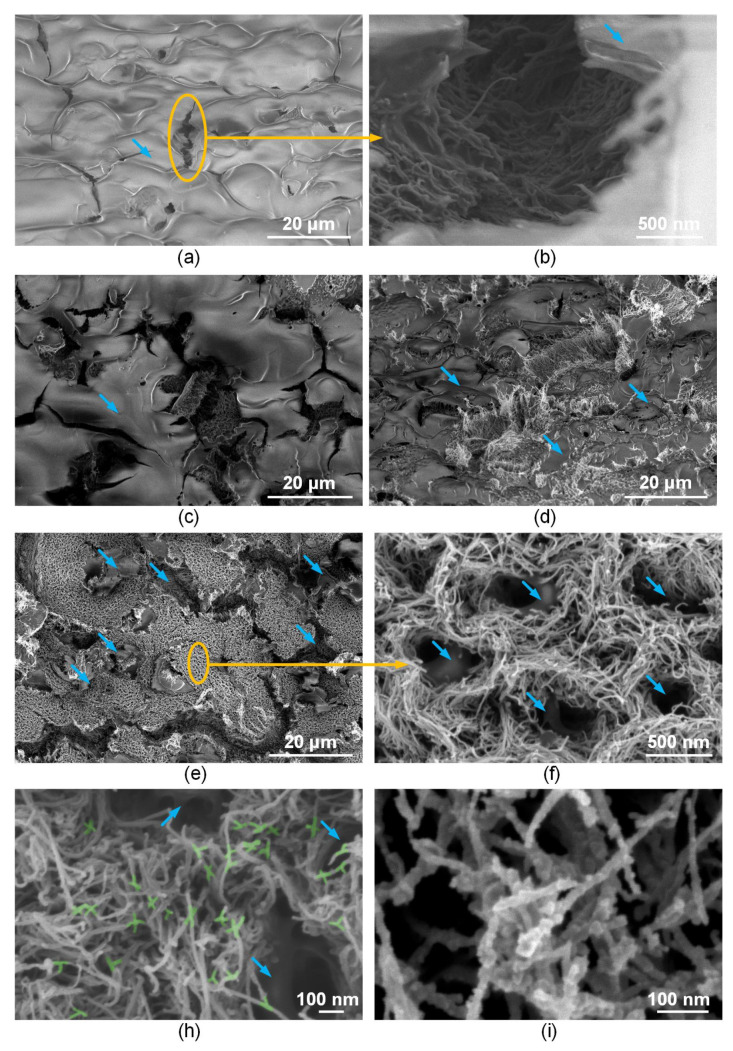
Interfaces based on MWCNT array on Si substrate (**a**,**b**), and interfaces formed under laser irradiation with an energy density of 0.011 (**c**), 0.013 (**d**), 0.015 (**e**–**h**), and 0.017 J/cm^2^ (**i**) (the blue arrows show the BSA). Nanotube binding regions are highlighted in green.

It is known that laser exposure to nanotubes leads to the formation of covalent bonds between carbon nanotubes [63]. C–C bonds are formed in the most radiation-heated defective regions of the nanotube backbone due to reduced thermal conductivity in these regions. Because of laser exposure, the synthesized vertical array of nanotubes (Figure 3b) changes its morphology, namely, on the one hand, the nanotube frame bends, and on the other hand, the nanotubes bind to each other (Figure 3d). That is, laser radiation with an energy density of 0.013 J/cm^2^ contributes to the formation of a nanotubes network in the array. Such MWCNT connections are highlighted in green in Figure 3d. It is also known that the formation of a scaffold structure is possible under the action of laser radiation, as was demonstrated in the case of scaffold based on single-walled carbon nanotubes in a biopolymer matrix of albumin and chitosan [53,64]. This formed structure provides increased electrical conductivity to the biopolymer material [55,65]. In this case, electrical conductivity strongly depends on the morphology of the structure created from polymers and a network of nanotubes [66]. In this regard, the morphology of interfaces based on MWCNT arrays coated with BSA biopolymer before and after laser exposure with different energy densities was analyzed using SEM (Figure 4).

Figure 4a shows an SEM image of an MWCNT array coated with a layer of BSA biopolymer. At the same time, nanotubes are not visible through the BSA layer. The biopolymer surface was characterized by the presence of rare cracks up to 30 µm in size. The study of cracks made it possible to detect nanotubes inside the biopolymer layer. However, nanotubes no longer had a vertical, but a chaotic orientation (Figure 4b). Thus, after synthesis, the nanotubes had a vertically oriented morphology. In addition, further hydrophilic treatment in plasma Ar+O_2_ was carried out, which contributes to the wetting of CNTs due to the formation of broken bonds [61]. When applying an aqueous suspension of BSA by spray-deposition, the flow was directed perpendicular to the substrate [67].

This flow leads to a change in the distance between the nanotubes and the manifestation of binding energies between individual nanotubes [68]. As a result, the nanotubes are reoriented, as well as their intertwining and, in some cases, the formation of bundles appeared [69]. In detail, the types of bonds and bond energies between CNTs and water in various variations are shown in the article [70].

Nanotubes were also coated with a biopolymer layer. The possibility of wrapping nanotubes with an albumin layer was described in [71]. Due to this, the nanotube diameter increased by 20–40%.

For the formation of interfaces, the energy density of pulsed laser exposure was experimentally established, which ensures the structuring of MWCNTs in the BSA matrix due to their binding to each other at the sites of defects. This process occurred at the energy density of 0.011–0.015 J/cm^2^. At a lower energy density, the binding of nanotubes in the array did not occur. The physical mechanism of the bonds formation between nanotubes can be explained as follows: during the process of laser exposure with an energy density of more than 0.011 J/cm^2^, energy is absorbed by electrons and converted into atomic energy. The collision of phonons with carbon atoms leads to the formation of defects such as vacancies and interstices in the nanotubes backbone due to the ballistic collision of electrons with carbon nuclei [48]. Due to the moderate temperature, the mobility of such defects leads to the breaking of C–C bonds in different MWCNT layers [72]. Chemical bonds are formed on the contact surface of bound carbon nanotubes. This leads to a surface reconstruction of the outer graphene layers and a decrease in their diameter. Crossed MWCNT joints are formed in the area of the outer graphene layer, which provides welding with the formation of heptagons and pentagons pairs of carbon atoms [73]. Figure 4c–e shows that when exposed to laser radiation, the amount of albumin on the sample’s surface decreases with an increase in energy density in the range 0.011–0.015 J/cm^2^.

It is also known that exposure to laser radiation on albumin leads to its ablation [74]. Figure 4f demonstrates that nanotubes in the BSA matrix formed a cellular structure. At the same time, X-, Y-, and T-shaped joints between nanotubes formed at 0.015 J/cm^2^, as Figure 4h shows. An increase in the energy density of more than 0.015 J/cm^2^ led to the destruction, related to carbon sublimation (Figure 4i) [75], of the samples structure and a decrease in electrical conductivity (Figure 4i).

The change in the concentration of chemical elements in the samples was estimated by the results of energy dispersion spectroscopy (EDX) (Table 2). This method allowed establishing the regularity of the change in the ratio of the chemical elements that make up albumin with an increase in the energy density of the affected laser radiation. An increase in the concentration of C atoms and a decrease in the number of N, O, and S atoms presented in the amino acids indicates a decrease for albumin in the samples after laser exposure.

**Figure 5:**
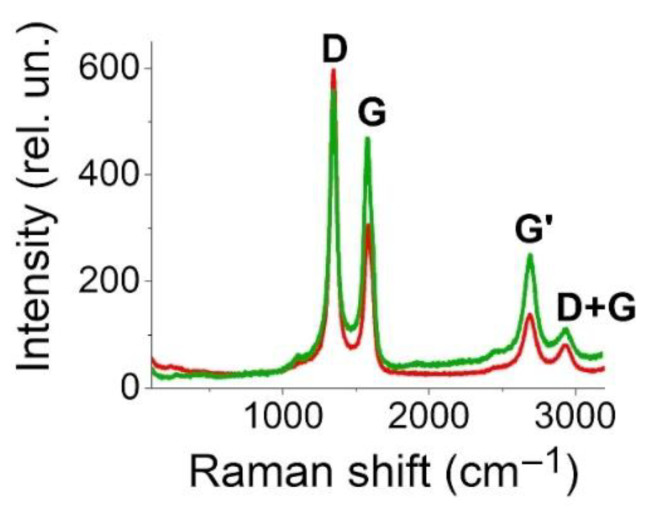
Raman spectroscopy of MWCNT arrays after synthesis (green line) and under irradiation with an energy density of 0.015 J/cm^2^ (red line).

It is known that MWCNTs have a large number of defects in the carbon structure [53]. The number of defects in MWCNT arrays may vary as a result of external influences. Therefore, in order to estimate the evolution of defectiveness after the laser structuring (the ratio of the D and G bands’ intensities lowered with a decrease in the defectiveness of the nanotubes [59]), a Raman spectroscopy of MWCNT arrays for both original and laser-structured samples at 0.015 J/cm^2^ was performed. The Raman spectra of the obtained samples include characteristic scattering bands G (~1585 cm^−1^) and D (~1350 cm^−1^), G’ (~2697 cm^−1^), and D+G (~2940 cm^−1^) (Figure 5). The ratio of D and G intensities in the original sample was 1.98, while after laser structuring, this ratio decreased to 1.21. Therefore, the laser influence leads to a decrease in the defectiveness of MWCNTs. A similar effect was observed in [76,77]. At the same time, the intensity of the ~1350 cm^–1^ peaks somewhat decreases after laser exposure, which indicates that the selected laser radiation energy density does not damage the main amount of MWCNTs [78]. The intensity of the G’ band (the overtone of the D band) decreased after laser treatment, which also indicated a decline in the defectiveness of the CNT [79].

Thus, the efficiency of the laser processing on the CNT arrays was established. As a result of the laser processing, we achieved a controlled structuring of nanotubes, especially at their upper ends.

### 3.2. Electrical Conductivity Measurements

Table 3 presents the results of measuring electrical conductivity of samples with and without laser exposure. It was found that when the ratio of BSA and MWCNT changed as a result of laser exposure, the electrical conductivity increased. At the same time, higher electrical conductivity indicates the effective formation of a network of bound nanotubes wrapped with albumin molecules. When exposed to a laser with an energy density of 0.015 J/cm^2^, the value of electrical conductivity increased by more than two times and amounted to 215.8 ± 10 S/m. A further increase in the radiation energy density to 0.017 J/cm^2^ contributed to a decrease in electrical conductivity. Therefore, interfaces for cellular studies were formed at 0.015 J/cm^2^.

In addition, according to the results of electrical conductivity and SEM, it can be concluded that the decrease in albumin concentration occurred with a gradient. The content of BSA mainly dropped at the maximum elevation, while closer to the base of the MWCNT array, a sufficient amount of BSA remained for the formation of cellular interfaces.

### 3.3. Fibroblast Cell Growth on Interfaces

The growth of fibroblast cells on laser-structured and unstructured samples with and without electrical stimulation was analyzed in the study. Electrical stimulation is a well-known method used to control cells by inducing changes in various cellular processes such as apoptosis, proliferation, differentiation, and migration [29,80]. This is due to the fact that all living cells have a transmembrane potential voltage difference, which is regulated by the balance between the accumulation of various ion concentrations both inside and outside the cell. The voltage difference of the transmembrane potential is controlled by ion channels and transporters [28]. The concentrations of Na^+^, K^+^, Ca^2+^, Mg^2+^, H^+^, and Cl^-^ ions are different inside and outside the cell [81]. K^+^ ions predominate inside the cell and Na^+^ ions predominate outside the cell. Hodgkin and Huxley’s research showed that due to the different concentrations of K^+^ ions on both sides of the membrane, energy is created to generate voltage on the membrane. Due to the uneven distribution of sodium ions, the activation of the cell reaction occurs; because of sodium ions, an active reaction of the cell becomes possible—an action potential. The high concentration of sodium ions on the outer side of the membrane is balanced by negatively charged chloride ions and the concentration of potassium is balanced by the presence of negatively charged anions, resulting in electrical neutrality inside and outside the cell.

Ion channels are responsible for the transfer of charged particles through the cell membrane [82]. Channels can be in open and closed states. The change of states is usually carried out because of changes occurring in the channel proteins, and is determined by factors coming both from outside (exogenous signals) and inside the cell (endogenous signals) [83]. In most cases, the state of an open or closed channel is characterized randomly. Despite this, there are signals that can change (increase or decrease) the permeability of the ion channel, and can also increase or decrease the probability of finding a structural protein configuration that promotes the transition between states [84].

The resting potential of most cells is characterized by a negative electric charge caused by a higher concentration of negative ions inside the cell compared to the concentration of negative ions outside the cell [85]. The negative resting potential creates a potential difference across the membrane that regulates voltage-sensitive ion transport and ATP synthesis in mitochondria. The negative charge is partly due to an excess of negative ions inside the cell (Donnan potential) and partly due to ionized groups on the membrane (surface potential). The use of electrical stimulation changes the transmembrane resting potential of the cell, which has a significant effect on cell functionality and cellular metabolism [86,87].

**Figure 6:**
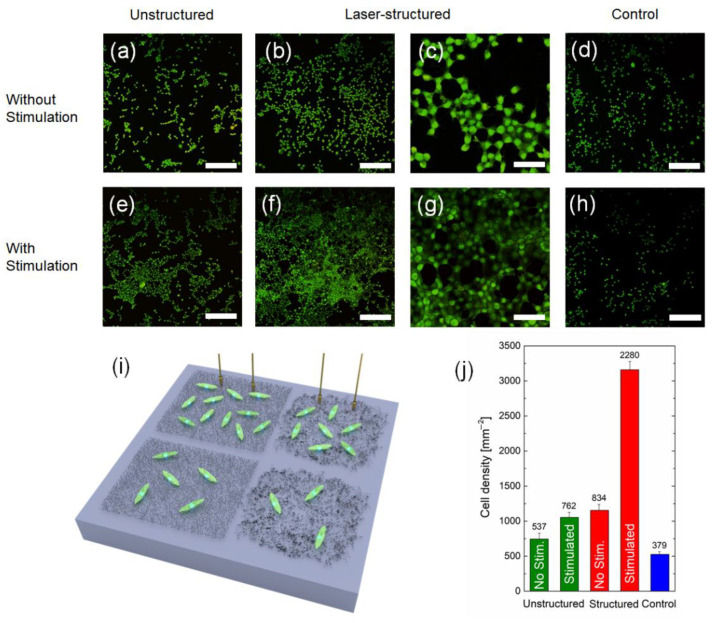
Examples of fluorescence microscope images of fibroblast cell cultures on Si substrates 24 h in vitro with unstructured (**a**,**e**) and laser-structured (**b**,**c**,**f**,**g**) MWCNT arrays with BSA immobilized with (**e**–**g**) and without (**a**–**c**) electrical stimulation and control samples (**d**,**h**). The scale bar for (**a**,**b**,**d**–**f**,**h**) is 200 µm, for (**c**,**g**) it is 50 µm. Schematic of fibroblasts grown on Si substrates with various conditions used in the experiment (**i**). The values on the bars in (**j**) represent the total number of live cells averaged over several areas of size 850 µm × 850 µm (0.7225 mm^2^). The results from three images on three different samples for each type of the condition were averaged for the statistics; n = 9.

For comparison, Si substrates (only chemically cleaned) were added as a reference. Figure 6 shows the density of living fibroblast cells obtained on Si substrates with MWCNT arrays, and the reference samples after 24 h in vitro (schematic image is given in Figure 6g). Figure 6a–f shows examples of fluorescence images obtained after live cell staining, whereas in Figure 6h, the statistics derived from these images are presented. The main results of this experiment were as follows:

- On laser-structured arrays, regardless of electrical stimulation, cells were grown more evenly and continuously distributed on the surface (i.e., homogeneous), whereas on samples without laser structuring, cells were distributed as separated “islands” (i.e., non-homogeneous).
- Electrical stimulation of cells on the structured MWCNT arrays brought a significant effect on the cell growth density, exceeding the ones without stimulation almost three times.

Among other biomedical applications, and due to their electrical conductivity and elastic properties, MWCNT-based composites can be used in the form of thin films, e.g., for a cell proliferation [53,88], as deformation sensors applied to a human skin [89], substrates for cells stimulation devices [90], or tissue regeneration [91]. Therefore, in order to demonstrate the potential of our MWCNT arrays for thin films biological applications, fibroblasts were immobilized on structured and unstructured arrays and electrical stimulation was applied. The fluorescence images (Figure 6a–h) show exemplary images of living fibroblasts cultured directly on the MWCNT arrays within 24 h. For the control (Figure 6d,h) samples, cell densities of ~500 mm^−2^ were obtained, which was comparable to standard values for fibroblast-coated substrates [92]. For unstructured arrays (Figure 6a,e), the density of cells increased for both nonstimulated (~740 mm^−2^) and stimulated (~1050 mm^−2^) samples in comparison to the control. The results showed that even for unstructured stimulated samples, the cell growth density increased by two times in comparison to control. The pronounced difference between structured and unstructured samples was that in case of structured arrays, cells were distributed more evenly than in the case of unstructured arrays, where cells tended to form clusters. In general, the ability of CNTs mixed with albumin to improve the cell adhesion is not novel [88]. However, our research is very interesting since it suggests that MWCNT-based composites on SiO_2_ are forming some sort of artificial docking sites for the better cell adhesion. It occurs since each CNT is a biocompatible defect on the surface preferable for cells to grow on [93]. Therefore, when the interface has a clustered array of MWCNTs, cells grow in a clustered form. On the other hand, when MWCNTs are distributed evenly via the laser structuring, homogeneous guided cell growth is observed. This underlines the significance of this work, because the MWCNT-based composites do not only seem to enable a tailoring of surface properties in terms of increasing the electrical conductivity, but also provide the ability to guide the cell distribution on the surface to use these properties in bioelectronics and biomedicine applications.

For structured arrays without electrical stimulation, there was not much difference in comparison to unstructured arrays with stimulation. The only observed distinction was an increase in the cell density for up to ~1150 mm^−2^. However, the major difference occurred in case of structured and electrically stimulated samples. In this case, the cell growth density increased up to ~3150 mm^−2^.

The ability of biocomposites to improve the cell adhesion and the influence of electrical stimulation are not novel [94,95]. However, this research suggests that structured MWCNT arrays on SiO_2_ allow forming highly conductive paths of aligned MWCNTs that result in enhanced influence of the electrical stimulation.

In other words, laser structuring distributed nanotubes evenly on the surface; due to this, cells were grown homogeneously and were more efficiently affected by stimulation currents. This underlines the significance of this work because cell stimulation on the structured CNT arrays provides the ability to use these properties in bioelectronics and biomedicine applications. Moreover, it is of great importance that MWCNT composites have a potential translatability into clinical applications.

### 3.4. Cardiomyocytes Cell Growth on CNT Arrays

The growth of cardiomyocytes cells on laser-structured and unstructured samples with and without electrical stimulation was analyzed similar to the previous experiment. This was performed to prove the influence of the CNT structuring and the electrical stimulation effect on the cell growth.

Previously, it was found that electrostimulation of cardiomyocytes was able to provide their synchronous beating instead of spontaneous. At the same time, the frequency of synchronous beat can also vary when the stimulation parameters change [96]. Each type of electrogenic cell (neurons, cardiomyocytes, etc.) has its own electrical signature in terms of amplitude and duration of the action potential. Moreover, due to its own impedance, the cell membrane allows both signal production and signal shielding [97].

**Figure 7:**
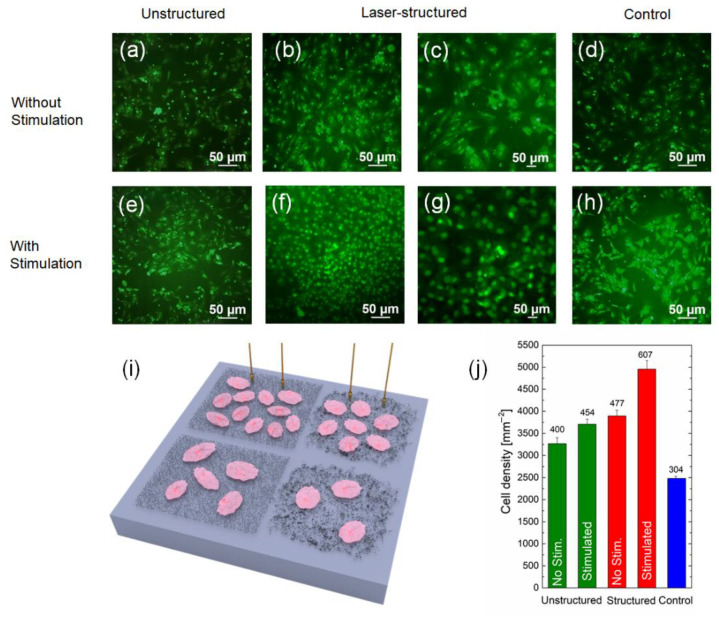
Examples of fluorescence microscope images of cardiomyocytes cell cultures on Si substrates 24 h in vitro with unstructured (**a**,**e**) and laser-structured (**b**,**c**,**f**,**g**) MWCNT arrays with BSA grown with (**e**–**g**) and without (**a**–**c**) electrical stimulation, and control samples (**d**,**h**). The scale bar for (**a**–**h**) is 50 µm. Schematic of cardiomyocytes grown on a Si substrate with various conditions used in the experiment (**i**). The values on the bars in (**j**) represent the total number of live cells averaged over several areas of size 350 µm × 350 µm (0.1225 mm^2^). The results from three images on three different samples for each type of the condition were averaged for the statistics; n = 9.

For comparison, Si substrates (only chemically cleaned) were added as a control. Figure 7 shows the density of living cardiomyocytes cells obtained on Si substrate with MWCNT arrays, and the reference samples after 24 h in vitro (schematic image is given in Figure 7i). Figure 7a–h show examples of fluorescence images obtained after live cell staining, whereas in Figure 7j, the statistics derived from these images are presented. The main results of this experiment were as follows:

- Cardiomyocytes growth rate was slower than in the case of fibroblasts; therefore, the initial cultivation period was increased to 4 days, and the time of electrical stimulation was reduced to 24 h, due to the high sensitivity of this type of cells to external influences.
- Laser structuring and electrical stimulation of cells together introduced a significant increase in the cell growth ratio similar to the previous experiment, which proved the efficacy of the combination of these methods for various biomedical applications.

In general, these results clearly follow the tendency for the fibroblasts experiments.

The fluorescence images (Figure 7a–h) show exemplary images of living cardiomyocytes cultured directly on the MWCNT arrays within 4 days. For the control (Figure 7d,h) samples, cell densities of ~2500 mm^−2^ were obtained. For unstructured arrays (Figure 7a,e), the density of cells increased for both nonstimulated (~3200 mm^−2^) and stimulated (~3700 mm^−2^) samples in comparison to the control. For structured arrays without electrical stimulation (~3900 mm^−2^), there was no significant difference in comparison to unstructured arrays with stimulation. A significant increase in the cell growth ratio was observed in the case of structured and electrically stimulated samples. It is known that nanoparticles increase the strength of the material, while the conductive nanoparticles give the materials the specific electrical conductivity necessary for the regeneration of conductive tissues, such as the heart and nerves. It was shown that electrically conductive composites with carbon nanotubes can be used for the proliferation of cardiac myoblasts [98] and for the promotion of myofibroblast transdifferentiation [99]. The cell growth density increased up to ~5000 mm^−2^. This proved the viability of using the combination of two methods: laser structuring of CNT arrays and electrical stimulation during cell growth. It is possible to suggest that this technology could represent a great tool for engineering surfaces for bioelectronics and biomedical purposes, such as improving cell adhesion or proliferation.

**Figure 8:**
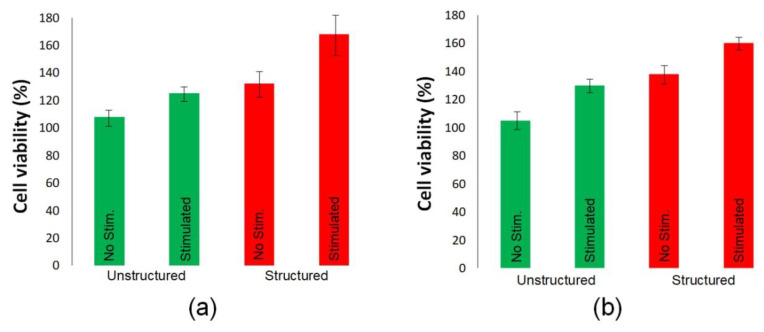
MTT assay of fibroblasts (**a**) and cardiomyocytes (**b**) cultures on Si substrates 24 h in vitro with unstructured and laser-structured MWCNT arrays with BSA grown with and without electrical stimulation compared to the control sample.

A similar relationship between the viability of fibroblasts and cardiomyocytes was obtained using MTT assay (Figure 8). For both types of cells, it was found that their maximum number was on laser-structured interfaces during electrical stimulation.

## 4. Conclusions

This study describes a way to tailor biocompatible nanostructures from arrays of multi-walled carbon nanotubes (MWCNTs) synthesized by the method of plasma-enhanced chemical vapor deposition from the gas phase on a silicon substrate. Nanostructures were created by changing the morphology of the MWCNT array in the albumin matrix simultaneously with the binding of nanotubes to each other using nanosecond pulsed laser radiation in the near-IR region of the spectrum (1064 nm). By applying the laser treatment, it was possible to decrease the defectiveness of the tubes, which was confirmed by Raman spectroscopy data. Using SEM, it was found that at a radiation energy density of 0.015 J/cm^2^, the optimal ratio of albumin and nanotubes in the samples was achieved, as well as the formation of a cellular structure and joints between nanotubes. Such a structure was characterized by a more than twofold increase in electrical conductivity (215.8 ± 10 S/m), as compared to the initial MWCNT array (100.7 ± 2 S/m).

Additionally, a setup that provides an electrical stimulation of cells was engineered. Using this setup, an effect of the electrical stimulation on variously tailored surfaces for fibroblasts and cardiomyocytes was stimulated and investigated. Finally, significant increase in the cell proliferation rate was achieved on structured arrays with electrical stimulation for both type of cell cultures.

This study indicates that laser-structured MWCNT arrays with biopolymer albumin coating might be a powerful tool for the improvement of cell experiments, bioelectronics devices, and even biomedical applications, ranging from biosensors to heart implants.

## Figures and Tables

**Table 1:** The synthesis parameters of MWCNT arrays.

Stage	T, °C	Time, min	Pr, torr	P, W (RF/LF)	Gas Phase				
					C_2_H_2_, (cm^3^/min)	Ar, (cm^3^/min)	NH_3_, (cm^3^/min)	H_2_, (cm^3^/min)	O_2_, (cm^3^/min)
Oxidation	280	10	3	100/0	-	100	-	-	100
Reduction	680	10	1.5	100/0	-	100	100	100	-
Synthesis	680	2	2	20/30	100	300	30	100	-
Hydrophilic treatment	280	0.3	1	0/20	-	100	-	-	100

**Table 2:** Results of EDX measurements.

Element	Content in Initial Sample (%) (Figure 4a,b)	Content in Samples after Laser Treatment (%)		
		0.011 J/cm^2^ (Figure 4c)	0.013 J/cm^2^ (Figure 4c)	0.015 J/cm^2^ (Figure 4e,f)
C	70.7 ± 0.6	72.6 ± 0.8	75.4 ± 0.8	80.0 ± 0.7
N	16.3 ± 0.5	14.7 ± 0.6	12.6 ± 0.4	10.2 ± 0.5
O	8.6 ± 0.2	8.0 ± 0.3	7.7 ± 0.3	6.3 ± 0.2
S	2.6 ± 0.1	2.4 ± 0.1	1.8 ± 0.1	1.3 ± 0.3
Na	1.7 ± 0.1	1.5 ± 0.1	1.4 ± 0.1	1.2 ± 0.2

**Table 3:** Electrical conductivity of samples with and without laser exposure.

	Initial Sample	Samples after Laser Exposure			
		0.011 J/cm^2^	0.013 J/cm^2^	0.015 J/cm^2^	0.017 J/cm^2^
Specific conductivity value (S/m)	100.7 ± 2	155.3 ± 4	174.8 ± 5	215.8 ± 10	112.2 ± 12

## Data Availability

The data presented in this study are available on request from the corresponding author.
